# Supplementary material for: Physician payment models and cardiac imaging in patients at low cardiovascular risk: A population-based cohort study in Alberta, Canada
Source: PLoS One. 2025 Nov 10;20(11):e0336399. doi: 10.1371/journal.pone.0336399 (PMC12599953; doi:10.1371/journal.pone.0336399)
Supplement: S1 Table — (PDF) [file pone.0336399.s001.pdf]

S1 Table: List of consultation codes used to define physician index visits.

Codes type	Code	Description
**Consult claim codes**		
SOMB	03.08A	Comprehensive consultation
	03.04A	Comprehensive assessment of a patient's condition requiring a complete history, a complete physical examination appropriate to the physician's specialty, an appropriate record and advice to the patient
	03.03A	Comprehensive assessment of a patient's condition requiring a complete history, a complete physical examination appropriate to the physician's specialty, an appropriate record and advice to the patient
	03.01L(all)	Diagnostic interview and evaluation, unqualified
	03.07B	Repeat consultation
	03.03F	Repeat office visit or scheduled outpatient visit in a regional facility, referred cases only
	03.03FA	Prolonged repeat office or scheduled outpatient visit in a regional facility, referred cases only, full 15 minutes or portion thereof for the first call when only one call
	03.08I	Prolonged consultation or visit, full 15 minutes, or major portion there off or the first call when only one call is claimed
	03.07A	Minor consult
	03.01O	Physician to Physician secure E-Consultation, consultant
	03.03N	Comprehensive evaluation including completion of forms to determine capacity as defined by the Personal Directives Act (PDA)
	03.03N	Comprehensive evaluation including completion of forms to determine capacity as defined by the Personal Directives Act (PDA)
	03.04N	Home visit, 1st patient, 0700-1700 weekdays (use time modifier)
	03.04M	Pre-operative history and physical examination in relation to an insured service
**Diagnostic claims that were used to define cardiology diagnoses for index visits**		
Code type	Code	Description
ICD9	394	Diseases of mitral valve
	395	Diseases of aortic valve
	398.91	Rheumatic heart failure (congestive)
	402.01	Malignant hypertensive heart disease with heart failure
	402.11	Benign hypertensive heart disease with heart failure
	402.91	Congestive heart failure, unspecified
	404.01	Hypertensive heart and chronic kidney disease, malignant, with heart failure and with chronic kidney disease stage I through stage IV, or unspecified
	404.03	Hypertensive heart and chronic kidney disease, malignant, with heart failure and with chronic kidney disease stage V or end stage renal disease

	404.11	Hypertensive heart and chronic kidney disease, benign, with heart failure and with chronic kidney disease stage I through stage IV, or unspecified
	404.13	Hypertensive heart and chronic kidney disease, benign, with heart failure and chronic kidney disease stage V or end stage renal disease
	404.91	Hypertensive heart and chronic kidney disease, unspecified, with heart failure and with chronic kidney disease stage I through stage IV, or unspecified
	404.93	Hypertensive heart and chronic kidney disease, unspecified, with heart failure and chronic kidney disease stage V or end stage renal disease
	410	Acute myocardial infarction
	411	Other acute and subacute forms of ischemic heart
	412	Myocardial infarction
	413	Angina pectoris
	414	Other forms of chronic ischemic heart disease
	424	Other diseases of endocardium
	425	Cardiomyopathy
	426	Conduction disturbance
	427	Cardiac dysrhythmias
	428	Heart failure
	429	defined descriptions and complications of heart disease
	785	Symptoms involving cardiovascular system
	786	Symptoms involving respiratory system and other chest symptoms
	794.3	Cardiovascular
**Pacemaker / ICD / CRTs**		
Code type	Code	Description
SOMB	49.7A	insertion of AV sequential pacemaker
	49.7C	transthoracic pacemaker
	49.7D	Transvenous pacemaker, permanent
	49.7F	Insertion of AV sequential pacemaker, 2 lead
	49.7G	Insertion of AV sequential pacemaker, 3 lead
	49.7H	Insertion of AV sequential pacemaker, 4 lead
	49.7J	Implantation of automatic internal cardioverter defibrillator - single RV lead
	49.7JA	Single chamber (right ventricular) implantable cardioverter defibrillator, insertion, and testing
	49.7K	Implantation of automatic internal cardioverter defibrillator - atrial and right ventricular lead
	49.7KA	Dual chamber implantable cardioverter defibrillator insertion and testing
	49.7L	Implantation of automatic internal cardioverter defibrillator - right ventricular and left ventricular lead
	49.7LA	Cardiac resynchronization defibrillator insertion without atrial lead and testing

	49.7 M	Implantation of automatic internal cardioverter defibrillator - atrial, right ventricular and left ventricular leads
	49.7MA	Cardiac resynchronization defibrillator insertion and testing
**Arrhythmia**		
Code type	Code	Description
ICD-10	I47.1	Supraventricular tachycardia
	I45.6	Pre-excitation syndrome
	I47.9	Paroxysmal tachycardia, unspecified
	I49.8	Other specified cardiac arrhythmias
	I49.9	Cardiac arrhythmia, unspecified
	R00.00	Tachycardia, unspecified
	R00.2	Palpitations
	I48	Atrial Fibrillation / flutter
	I47.2	Ventricular tachycardia
	I49.01	Ventricular fibrillation
	I49.02	Ventricular flutter
	I46.9	Cardiac Arrest
