# Supplementary material for: Physician payment models and cardiac imaging in patients at low cardiovascular risk: A population-based cohort study in Alberta, Canada
Source: PLoS One. 2025 Nov 10;20(11):e0336399. doi: 10.1371/journal.pone.0336399 (PMC12599953; doi:10.1371/journal.pone.0336399)
Supplement: S2 Table — (PDF) [file pone.0336399.s002.pdf]

S2 Table. List of ICD9 codes used to define cardiovascular disease and cardiovascular risk factors.

Cardiac vascular disease		
	ICD codes	Source
MI	ICD10: I21*, I22*<br>ICD9: 410*<br>One DAD visit prior to study date with a permanent lookback period	Tonelli et al.
CHF	ICD10: I09.0, I25.5, I42.0, I42.5 – I42.9, I43, I50<br>ICD9: 398.91, 402.01, 402.11, 402.91, 404.01, 404.03, 404.11, 404.13, 404.91, 404.93, 425.4 – 425.9, 428<br>1 DAD or 2 claims in 2 years or less	Tonelli et al.
Stroke	ICD10: G45, H34.1, I60, I61, I63, I64<br>ICD9: 362.3, 430, 431, 433, 434, 435, 436<br>at least one claims, DAD or NACRS diagnosis prior to study date with a permanent lookback period	Tonelli et al.
PVD	ICD10: I70.2<br>ICD 9: 440.2<br>at least one claims, DAD or NACRS diagnosis prior to study date with a permanent lookback period	Tonelli et al.
CABG/ PCI	CABG: 1IJ76<br>PCI: 1IJ50, 1IJ57GQ<br><br>one hospital visits using CCI code in most responsible diagnosis or any one of the secondary diagnostic codes	https://www.ncbi.nlm.nih.gov/pmc/articles/PMC2950731/
IHD	ICD10: I20* – I25*<br>ICD9: 410* – 414*<br>One year prior to study date<br><br>two physician billing codes (with one of the physician billing codes being from a specialist or general practitioner/family physician in a hospital or emergency room setting) or one hospital discharge abstract (any position)	https://www.ncbi.nlm.nih.gov/pmc/articles/PMC2950731/
Valvular heart disease	Mitral or aortic disease (ICD-9 394, 395, 396, 424.0, 424.1 or ICD-10 I05, I06, I08.0, I08.1, I085.2, I08.3, I34, I35)<br>Tricuspid or pulmonary valvular disease (ICD-9 397, 424.2, 424.3, ICD-10 I07, I08.1, I08.2, I08.8, I08.9, I36, I37)	

	Valve surgery (ICD-9 35.0, 35.1, 35.2, 35.96, 35.97, 35.99 and ICD-10 (CCI) code 1.HS.80,1.HS.90, 1.HT.80, 1.HT.89, 1.HT.90, 1.HU.80, 1.HU.90, 1.HV.80, 1.HV.90)<br><br>At least one claim or one DAD using ICD codes in most responsible diagnosis or any one of the secondary diagnostic codes with a permanent lookback period	
Traditional risk factors		
Diabetes	ICD 10: 250<br>ICD 9: E10 – E14<br>One DAD or two claims in 2 years or less	Tonelli et al.
Dyslipidemia<br>Chol	LDL>=3.5<br>(average of 5 years calculations)	https://pubmed.ncbi.nlm.nih.gov/31170925/<br><br>https://bmcnephrol.biomedcentral.com/articles/10.1186/s12882-019-1351-9
	one statin prescription (in past year)	https://pubmed.ncbi.nlm.nih.gov/34923023/
Hypertension	ICD 10: 401 402 403 404 405<br>ICD 9: I10 I11 I12 I13 I15<br>One DAD or two NACRS in 2 years or less	Tonelli et al.
Age	men >=50 and hypertension	
	women >=60 and hypertension	
CKD	eGFR<60 (one uncalibrated measure closest to index date. If two measures on the same day, take the highest measure) or proteinuria (albuminuria A3)	
Treatment Anatomical classification codes (ATC - statin or other lipid-lowering therapy).		
Statins, statins with other combinations	C10AA01, C10AA02, C10AA03, C10AA04, C10AA05, C10AA06, C10AA07, C10BX01, C10BX02, C10BX03, C10AB01, C10AB02, C10AB04, C10AB05	simvastatin, lovastatin, pravastatin, fluvastatin, atorvastatin, cerivastatin, rosuvastatin, acetylsalicylic acid /simvastatin, acetylsalicylic acid /pravastatin, atorvastatin/amlodipine, clofibrate, bezafibrate, gemfibrozil, fenofibrate

Fibrates	C10AB01, C10AB02, C10AB04, C10AB05, C10AC01, C10AC02, C10AC04	clofibrate, bezafibrate, gemfibrozil, fenofibrate, Colestyramine, colestipol, colesevelam
Bile acid sequestrants	C10AD02	Nicotinic acid
Nicotinic acid and derivatives	C10AX01, C10AX02, C10AX06, C10AX09, C10AX13, C10AX14	dextrothyroxine sodium, probucol, omega-3-triglycerides included other esters and acids, ezetimibe, evolocumab, alirocumab
