# Supplementary material for: Physician payment models and cardiac imaging in patients at low cardiovascular risk: A population-based cohort study in Alberta, Canada
Source: PLoS One. 2025 Nov 10;20(11):e0336399. doi: 10.1371/journal.pone.0336399 (PMC12599953; doi:10.1371/journal.pone.0336399)
Supplement: S3 Table — (PDF) [file pone.0336399.s003.pdf]

S3 Table. Procedure codes used to define cardiac testing.

**Echocardiography**	
X306A	Complex Complete Echocardiogram
X306B	Non-Complex Complete Echocardiogram
X307	Ultrasound, heart, Echocardiogram, limited
02.82A	Comprehensive diagnostic trans-esophageal echocardiography
03.44A	Physician personal and continuous monitoring during the provision of dobutamine infusion for the purposes of pharmacologic stress imaging
**Myocardial Perfusion Imaging (Nuclear)**	
X170	Thallium myocardial perfusion imaging (rest study)
X171	Thallium myocardial perfusion imaging (rest and exercise)
X172	Gated cardiac imaging (rest study)
X173	Gated cardiac imaging (rest and exercise)
03.41D	Intravenous dipyridamole administration for thallium imaging, professional component only
03.44A	Physician personal and continuous monitoring during the provision of dobutamine infusion for the purposes of pharmacologic stress imaging
**Exercise Treadmill Testing**	
03.41A	Maximal stress electrocardiogram, with or without pulse oximetry, technical only
03.41B	Interpretation
03.41C	Continuous personal physician monitoring, with or without pulse oximetry
