# Supplementary material for: Physician payment models and cardiac imaging in patients at low cardiovascular risk: A population-based cohort study in Alberta, Canada
Source: PLoS One. 2025 Nov 10;20(11):e0336399. doi: 10.1371/journal.pone.0336399 (PMC12599953; doi:10.1371/journal.pone.0336399)
Supplement: S4 — (PDF) [file pone.0336399.s004.pdf]

S4 Table. Demographic characteristics of people who received and did not receive cardiac imaging.

	No cardiac imaging (n=288,767)	Received at least one non-invasive cardiac imaging test (n=109,328)	P-value
Overall	72.5 (288,767)	27.5 (109,328)	398,095
**Age, % (n)**			
<=40	81.7 (153,801)	18.3 (34,361)	<0.001
41-60	64.8 (108,516)	35.2 (58,963)	
61-80	60.8 (22,804)	39.2 (14,709)	
>81	69.5 (1,632)	30.5 (715)	
Missing	77.6 (2,014)	22.4 (580)	
**Sex, % (n)**			
Male	68.4 (115,755)	31.6 (53,373)	<0.001
Female	75.5 (170,998)	24.5 (55,374)	
Undefined	0.0 (0)	100.0 (1)	
Missing	77.6 (2,014)	27.5 (580)	
**Comorbidities, % (n)**			
0 comorbidity	71.4 (145,545)	28.6 (58,370)	<0.001
1 comorbidity	72.4 (88,663)	27.6 (33,850)	
2 comorbidities	74.7 (35,658)	25.3 (12,071)	
>=3 or 4 comorbidities	78.5 (16,956)	21.5 (4,635)	
5 or more comorbidities	82.9 (1,945)	17.1 (402)	
**Income quintile, % (n)**			
Lowest	76.9 (73,381)	23.1 (22,042)	<0.001
2nd	73.3 (60,054)	26.7 (21,876)	
3rd	71.6 (53,203)	28.4 (21,132)	
4th	70.8 (49,869)	29.2 (20,554)	
Highest	68.2 (47,676)	31.8 (22,268)	
Undefined	75.9 (4,584)	24.1 (1,456)	
**Rurality, % (n)**			
Urban	72.3 (261,698)	27.7 (100,366)	<0.001
Rural	75.0 (23,658)	25.0 (7,898)	
Missing	76.2 (3,411)	23.8 (1,064)	
**Patient attachment to GPs, % (n)**			
75-100	67.5 (72,740)	32.5 (34,836)	<0.001
50 - <75	71.4 (93,570)	28.6 (37,415)	
<50	76.5 (113,803)	23.5 (34,921)	
Missing	80.5 (8,924)	19.5 (2,156)	
**Physician characteristics**			
**Age, mean (SD)**	45.0 (10.0)	46.8 (9.7)	

**Age, % (n)**			
<=40	76.6 (110,114)	23.4 (33,706)	<0.001
40 - 60	70.3 (154,567)	29.7 (65,223)	
>=60	70.9 (23,801)	29.0 (9,731)	
Missing	29.9 (285)	70.1 (668)	
**Payment model and speciality, % (n)**			
Salary-based Cardiologist	81.4 (4,382)	18.6 (1,001)	<0.001
Salary-based Internal medicine specialists	95.6 (33.594)	4.4 (1,547)	<0.001
FFS Cardiologist	26.7 (15,508)	73.3 (42,661)	<0.001
FFS Internal medicine specialists	78.6 (235,283)	21.4 (64,119)	<0.001
**Gender, % (n)**			
Female	80.3 (67,905)	19.7 (16,613)	<0.001
Male	70.6 (220,649)	29.4 (92,048)	
Missing	24.2 (213)	75.8 (667)	
**Country of training, % (n)**			
Canada	75.9 (150,238)	21.2 (47,827)	<0.001
High/upper middle income countries with similar medical training system to Canada**	58.2 (21,601)	41.8 (15,510)	
Other high/upper middle-income country***	66.9 (61,574)	33.1 (30,416)	
Lower middle/low-income country***	79.4 (53,906)	20.6 (13,983)	
Unknown	47.6 (1,448)	52.4 (1,592)	
**Clinical Workload, % (n)**			
<15%	85.1 (15,439)	14.9 (2,679)	<0.001
15-30%	74.9 (76,142)	25.1 (25,467)	
>30%	70.8 (197,246)	29.2 (81,181)	
Missing	96.8 (30)	3.2 (1)	
**Years in practice, % (n)**			
>15	77.5 (99,727)	22.5 (28,948)	<0.001
15-30	70.9 (140,219)	29.7 (57,456)	
>30	69.1 (47,976)	30.9 (21,417)	

Missing	35.9 (849)	64.1 (1,507)	
**Patient volume, % (n)**			
4 (highest)	69.0 (152,895)	31.0 (68,794)	<0.001
3	72.4 (67,513)	27.6 (25,702)	
2	81.2 (45,831)	18.8 (10,611)	
1 (lowest)	84.2 (22,528)	15.8 (4,221)	
**Zone, % (n)**			
Urban 1	62.4 (92,123)	37.6 (55,404)	<0.001
Urban 2	77.0 (149,723)	23.0 (44,696)	
Rural 1	80.0 (13,838)	20.0 (3,463)	
Rural 2	83.6 (14,533)	16.4 (2,842)	
Rural 3	86.4 (18,514)	13.6 (2,921)	
Missing	72.5 (46)	4.2 (2)	
