# Supplementary material for: Physician payment models and cardiac imaging in patients at low cardiovascular risk: A population-based cohort study in Alberta, Canada
Source: PLoS One. 2025 Nov 10;20(11):e0336399. doi: 10.1371/journal.pone.0336399 (PMC12599953; doi:10.1371/journal.pone.0336399)
Supplement: S5 Table — (PDF) [file pone.0336399.s005.pdf]

S5 Table. Explained and unexplained physician, patient, and geographic-level variation across models estimating odds of cardiac testing.

**Variance Partition Coefficient**									
Variable	Two-level unconditional model			Three-level unconditional model*			Three-level patient and patients characteristics model*		
	coefficient (95 CI%)	OR (95% CI)	p-value	coefficient (95 CI%)	OR (95% CI)	p-value	coefficient (95 CI%)	OR (95% CI)	p-value
Intercept	-2.09 (-2.24, -1.94)	-0.12 (0.11, 0.14)	<0.001	-2.28 (-2.48, -2.07)	0.10 (0.083, 0.13)	<0.001	-1.60 (-2.25, -0.95)	0.20 (0.02 – 0.39)	<0.001
Variance of random effects									
τ^2 -physician	3.86 (3.44, 4.33)			2.79 (2.44, 3.18)			1.88 (1.60 – 2.15)		
τ^2 -site				1.291 (0.96, 1.73)			0.98 (0.73 – 1.31)		
PCV	reference			0.28			0.51		
VPC/ ICC - physician	0.54			0.38			0.31		
VPC/ ICC -site				0.18			0.16		
MOR - physician	6.52			4.92			3.70		
MOR -zone				2.96			2.57		

OR: odds ratio, PCV: proportional change of the variance, VPC: variance partition coefficient, ICC: intra class correlation, MOR: median odds ratio

*physicians and sites are crossed in three-level model
