# Supplementary material for: Physician payment models and cardiac imaging in patients at low cardiovascular risk: A population-based cohort study in Alberta, Canada
Source: PLoS One. 2025 Nov 10;20(11):e0336399. doi: 10.1371/journal.pone.0336399 (PMC12599953; doi:10.1371/journal.pone.0336399)
Supplement: S6 Table — (PDF) [file pone.0336399.s006.pdf]

S6 Table. Scenarios highlighting variation in likelihood of cardiac testing across different physician groups.

	Unadjusted (basecase)	Scenario 1*	Scenario 2**	Scenario 3***	Scenario 4****
Salary-based internal medicine specialists	4.4%	9.0%	6.7%	12.1%	7.8%
FFS internal medicine specialists	21.4%	14.8%	11.1%	19.5%	12.9%
Salary-based cardiologists	18.6%	32.6%	25.9%	40.3%	29.2%
FFS cardiologist	73.3%	64.1%	56.3%	71.3%	60.4%

*Scenario 1: Male; income quintile “3”; patient age 41-60; fewer than 3 patient non-cardiac comorbidities; physician gender - man; physician age 50; physician zone – urban 1

**Scenario 2: Male; income quintile "3"; patient age 41-60; fewer than 3 patient non-cardiac comorbidities; physician gender-Woman; physician age 50; physician zone – urban 1

***Scenario 3: Male; income quintile “3”; patient age 41-60; fewer than 3 patient non-cardiac comorbidities; physician gender-man; physician age 50; physician zone – rural 3

****Scenario 4: Female; income quintile "3"; patient age 41-60; fewer than 3 patient non-cardiac comorbidities; physician gender-man; physician age 50; physician zone – urban 1
