# Supplementary material for: Physician payment models and cardiac imaging in patients at low cardiovascular risk: A population-based cohort study in Alberta, Canada
Source: PLoS One. 2025 Nov 10;20(11):e0336399. doi: 10.1371/journal.pone.0336399 (PMC12599953; doi:10.1371/journal.pone.0336399)
Supplement: Attachment — Submitted filename: Response to reviewers PLOS Sept 2.docx [file pone.0336399.s008.docx]

**Journal Requirements:**

When submitting your revision, we need you to address these additional requirements.

1. Please ensure that your manuscript meets PLOS ONE's style requirements, including those for file naming. The PLOS ONE style templates can be found at <https://journals.plos.org/plosone/s/file?id=wjVg/PLOSOne_formatting_sample_main_body.pdf> and <https://journals.plos.org/plosone/s/file?id=ba62/PLOSOne_formatting_sample_title_authors_affiliations.pdf>

**Thank you. We have done so.**

2. PLOS requires an ORCID iD for the corresponding author in Editorial Manager on papers submitted after December 6th, 2016. Please ensure that you have an ORCID iD and that it is validated in Editorial Manager. To do this, go to ‘Update my Information’ (in the upper left-hand corner of the main menu), and click on the Fetch/Validate link next to the ORCID field. This will take you to the ORCID site and allow you to create a new iD or authenticate a pre-existing iD in Editorial Manager.

**Thank you. It appears that I had multiple records in PLOS but that has been corrected, and my ORCID number now appears.**

3. We note that you have indicated that there are restrictions to data sharing for this study. For studies involving human research participant data or other sensitive data, we encourage authors to share de-identified or anonymized data. However, when data cannot be publicly shared for ethical reasons, we allow authors to make their data sets available upon request. For information on unacceptable data access restrictions, please see <http://journals.plos.org/plosone/s/data-availability#loc-unacceptable-data-access-restrictions>.

Before we proceed with your manuscript, please address the following prompts:

a) If there are ethical or legal restrictions on sharing a de-identified data set, please explain them in detail (e.g., data contain potentially identifying or sensitive patient information, data are owned by a third-party organization, etc.) and who has imposed them (e.g., a Research Ethics Committee or Institutional Review Board, etc.). Please also provide contact information for a data access committee, ethics committee, or other institutional body to which data requests may be sent.

b) If there are no restrictions, please upload the minimal anonymized data set necessary to replicate your study findings to a stable, public repository and provide us with the relevant URLs, DOIs, or accession numbers. Please see <http://www.bmj.com/content/340/bmj.c181.long> for guidelines on how to de-identify and prepare clinical data for publication. For a list of recommended repositories, please see <https://journals.plos.org/plosone/s/recommended-repositories>. You also have the option of uploading the data as Supporting Information files, but we would recommend depositing data directly to a data repository if possible.

Please update your Data Availability statement in the submission form accordingly.

**See below**

4. In the online submission form, you indicated that we cannot make our dataset available to other researchers due to our contractual arrangements with the provincial health ministry (Alberta Health), who is the data custodian. Researchers may make requests to obtain a similar dataset at <https://absporu.ca/research-services/service-application/>.

All PLOS journals now require all data underlying the findings described in their manuscript to be freely available to other researchers, either 1. In a public repository, 2. Within the manuscript itself, or 3. Uploaded as supplementary information.
This policy applies to all data except where public deposition would breach compliance with the protocol approved by your research ethics board. If your data cannot be made publicly available for ethical or legal reasons (e.g., public availability would compromise patient privacy), please explain your reasons on resubmission and your exemption request will be escalated for approval.

**I corresponded with Dr Lin on this matter on June 24 (response from Ethan Krajewski, Peer Review Operations Specialist, suggesting our response was satisfactory). I will reiterate that here.**

**“There are legal restrictions on sharing our dataset, even fully de-identified data, from our provincial health ministry, who is the formal custodian for the health administrative data that we use for this study. We have been advocating for a change to their policy, which is embedded within the contract through which we access the health data, but have not been successful. Attached, please see a letter from the Alberta government health ministry confirming this legal restriction, and that we would be in breach of the law if we were to make a de-identified dataset available.**

**As an alternative, those interested in conducting similar analyses can make requests to obtain a similar dataset at https://absporu.ca/research-services/service-application/ ." The data they receive would allow them to replicate the analyses we have performed for this work.**

**As you note above, we have informed readers of this opportunity, and have modified our statement to make it clear they could replicate our analyses.**

**Unfortunately, this policy doesn’t just apply to this one study – it applies to all research conducted by our group using this dataset. Of note, this rationale was recently accepted by PLOSOne within the past year -** [**https://pmc.ncbi.nlm.nih.gov/articles/PMC11156280/**](https://pmc.ncbi.nlm.nih.gov/articles/PMC11156280/) **using the same Data Sharing statement. Thank you very much for considering this exemption.**

5. Thank you for stating the following financial disclosure:
A Canadian Institutes of Health Research Foundation Grant to Manns and a Data Collection Grant from the Clinical Research Fund, University of Calgary to Ogundeji and Manns funded this study

Please state what role the funders took in the study. If the funders had no role, please state: "The funders had no role in study design, data collection and analysis, decision to publish, or preparation of the manuscript."
If this statement is not correct you must amend it as needed.
Please include this amended Role of Funder statement in your cover letter; we will change the online submission form on your behalf.

**Thank you. I can confirm that “The funders had no role in study design, data collection and analysis, decision to publish, or preparation of the manuscript." This has now been added.**

6. Please remove all personal information, ensure that the data shared are in accordance with participant consent, and re-upload a fully anonymized data set.

Note: spreadsheet columns with personal information must be removed and not hidden as all hidden columns will appear in the published file.

Additional guidance on preparing raw data for publication can be found in our Data Policy (<https://journals.plos.org/plosone/s/data-availability#loc-human-research-participant-data-and-other-sensitive-data>) and in the following article: <http://www.bmj.com/content/340/bmj.c181.long>.

**See response above. Unfortunately, we are not able to share our de-identified data.**

7. Please include captions for your Supporting Information files at the end of your manuscript, and update any in-text citations to match accordingly. Please see our Supporting Information guidelines for more information: <http://journals.plos.org/plosone/s/supporting-information>.

**See response above. Unfortunately, we are not able to share our de-identified data.**

[Note: HTML markup is below. Please do not edit.]

Reviewers' comments:

Reviewer's Responses to Questions

**Comments to the Author**

1. Is the manuscript technically sound, and do the data support the conclusions?

The manuscript must describe a technically sound piece of scientific research with data that supports the conclusions. Experiments must have been conducted rigorously, with appropriate controls, replication, and sample sizes. The conclusions must be drawn appropriately based on the data presented.

Reviewer #1: Yes

Reviewer #2: Partly

2. Has the statistical analysis been performed appropriately and rigorously?

Reviewer #1: Yes

Reviewer #2: Yes

3. Have the authors made all data underlying the findings in their manuscript fully available?

The [PLOS Data policy](http://track.editorialmanager.com/CL0/http:%2F%2Fwww.plosone.org%2Fstatic%2Fpolicies.action%23sharing/1/010f01975cb24240-bfe1730f-4c20-441d-84e6-4b8e4ea40628-000000/irxFHAFfyR2mfCBk4hU5aIjhPEWs87SFAkaI-fVys9k=215) requires authors to make all data underlying the findings described in their manuscript fully available without restriction, with rare exception (please refer to the Data Availability Statement in the manuscript PDF file). The data should be provided as part of the manuscript or its supporting information, or deposited to a public repository. For example, in addition to summary statistics, the data points behind means, medians and variance measures should be available. If there are restrictions on publicly sharing data—e.g. participant privacy or use of data from a third party—those must be specified.

Reviewer #1: Yes

Reviewer #2: Yes

4. Is the manuscript presented in an intelligible fashion and written in standard English?

PLOS ONE does not copyedit accepted manuscripts, so the language in submitted articles must be clear, correct, and unambiguous. Any typographical or grammatical errors should be corrected at revision, so please note any specific errors here.

Reviewer #1: Yes

Reviewer #2: Yes

**Thank you**

5. Review Comments to the Author

Please use the space provided to explain your answers to the questions above. You may also include additional comments for the author, including concerns about dual publication, research ethics, or publication ethics. (Please upload your review as an attachment if it exceeds 20,000 characters)

Reviewer #1: This paper is well-written, clearly structured, and addresses an important issue in health services research. While the study provides valuable insights, the following suggestions are intended to further strengthen its quality and enhance its potential for publication.

• The study addresses a relevant and timely topic in health services research by examining how physician payment models influence cardiac imaging in low-risk populations.
• The use of a large retrospective cohort from administrative health data in Alberta enhances the robustness and generalizability of the findings.
• The manuscript is generally well-written, with clear articulation of the research objective, methods, results, and conclusions.
• The study design is appropriate for the research question, and the use of multilevel, multivariable logistic regression is methodologically sound.
• The classification of patients into low cardiovascular risk is appropriately done using administrative data, although the limitations of such classification should be more clearly acknowledged.
• The description of the payment models (fee-for-service vs. salary-based) could benefit from additional context to aid readers unfamiliar with the Canadian healthcare system.
• The finding that salary-based physicians, particularly internal medicine specialists, are less likely to order imaging is compelling and aligns with hypotheses regarding financial incentives.
• The discussion provides a good synthesis of the results, although more emphasis could be placed on potential policy implications of these findings.
• The conclusion is supported by the presented data and underscores the role of physician incentives in potentially unnecessary care.
• The authors performed several sensitivity analyses to confirm the robustness of their findings, which is commendable.
• The manuscript could improve by including a brief explanation of the ethical approval process, particularly given the use of administrative health data.

**Thank you. We have provided additional detail.**

• Some figures or tables (if available in the full version) may need more detailed legends to stand alone for clarity.

**Thank you. We have modified several of the tables / figures based on reviewers’ comments and added additional detail in the legends**

• There is no indication of ethical concerns or misconduct related to research design, data analysis, or authorship.

Reviewer #2: Good work,the topic is timely and relevant, addressing an underexplored area within health services research. However, the manuscript would benefit from several improvements to maximize its global relevance, contextual clarity, and analytical completeness. The comments below are intended to guide your revision constructively.

1. Title and Keywords
1.1 The title is technically accurate but long and clinical. Consider simplifying it to improve accessibility, e.g., Physician Payment Models and Cardiac Imaging in Low-Risk Patients: A Population-Based Cohort Study in Alberta, Canada.

**Thank you. We have accepted your suggestion (with one small wording change)**

1.2 The keywords do not sufficiently capture the paper’s scope or uniqueness. Suggested replacements: physician remuneration, cardiac test overuse, primary care incentives, Alberta health system, low-value care.

**Thank you. We have added physician remuneration, cardiac test overuse, low value care.**

2. Abstract
2.1 Avoid jargon like “receipt of imaging” — use more universally understood terms.

**Thank you. This has been changed to cardiac imaging throughout the manuscript.**

2.2 The conclusion is too narrowly framed. Consider broadening its relevance to readers unfamiliar with Canadian payment models.

**Thank you. We have broadened the conclusion, while keeping in mind that many PLOS ONE readers will be international**

3. Introduction
3.1 The introduction is well-focused on Choosing Wisely and overuse but lacks global context. A comparison of physician payment models across countries (e.g., FFS, capitation, salary) would help orient an international audience.

**An additional paragraph has been included in the introduction to include relevant literature across broader health systems context (see pages 3 and 4).**

3.2 The literature cited is mainly Canadian; expanding to include studies from the US, UK, or Australia would strengthen framing. broaden this

**An additional paragraph has been included in the introduction to include relevant literature across broader health system contexts (see pages 3 and 4). We’ve also augmented the evidence cited to include other countries.**

3.3 The study objective is clearly stated but could be linked more explicitly to the broader issue of health system design and policy reform.

**In addition to linking the study to global contexts, we’ve included additional text on the potential influence/impact on physician payment policy reforms**

4. Methods
4.1 The study design is appropriate, but the term “retrospective cohort” may overstate longitudinal rigor. A more precise description would be: “A population-based cohort study using health service data in Alberta, Canada.”

**The title and description have been changed to reflect this.**

4.2 The assumption of imaging attribution to the nearest outpatient visit should be explained and justified.

**Thank you. We are happy to expand, and have added some additional text to the limitation section of the paper. Without clinical data that indicated the ordering physician, we examined the distribution of imaging tests 90 days before patients’ initial visits and up to a year following their initial visits (see figure below). We determined that the majority of tests were ordered during the -30 to +90 day window (Figure). We also spoke to cardiologists about their test ordering practices. Some cardiology specialists routinely ordered testing in advance of their new visits while others ordered them afterwards. Given that there is a short wait time (<30 days) for cardiology imaging studies in Alberta, but often long waits to have a consultation with a specialist, we made the assumption that tests ordered within the 30 days before or 90 days after were related to the clinic visit.**

**We cannot be certain that the tests were ordered by the internist or cardiologist, but it seems unlikely that primary care physicians would change their own imaging ordering practices based on the payment model of the specialist they are referring patients to. So if primary care physicians were ordering many of the tests, we would expect this to dilute the observed association.**

4.3 The impact of COVID-19 on test volumes in 2020 is not discussed. A sensitivity analysis excluding 2020 or a clear rationale for inclusion is needed.

**Our study period did not extend into the period of the pandemic.**

4.4 While physician- and patient-level variables are handled well, missing clinical variables (e.g., symptoms) should be flagged more explicitly in Methods, not just in Discussion.

**Thank you. Please see pages 8 and 9 for additional clarification. “Fourth, because we did not have data on the patients’ presenting symptoms or physical findings, we did a fourth sensitivity analysis excluding patients who were diagnosed with cardiovascular disease, arrythmia, or required a pacemaker over the subsequent year.”**

4.5 Ethics approval is mentioned but would benefit from more clarity — include board name, reference number, or waiver status.

**Thank you. We have made additional changes to this section.**

5. Results
5.1 Results are presented clearly but are overly text-heavy. Consider including:
A forest plot of ORs **–**

**This is an excellent suggestion and we have changed Tables 2 and 3 to figures displaying Forest plots (Figure 3 and 4).**

A STROBE-style flow diagram for cohort construction-

**We agree this is critical. We had inadvertently included this as an additional file, so while it was present, we suspect the reviewer may have missed this. We have now included within the paper, and in an additional file.**

A bar or line chart showing temporal trends (2012–2020) –

**We are not entirely sure what the reviewer is suggesting in terms of temporal trends over time, but we assumed it was a temporal trend for our main exposure – payment model. To explore this we performed additional analyses:**

**1. Examine** **risk of testing by year over the entire time period, and by type of physician (we show this in a tabular and then a figure format). This shows that the overall trend over time is similar.**

|  | FFS CARD | AMHSP CARD | FFS INMD | AMHSP INMD | Overall |
| --- | --- | --- | --- | --- | --- |
| 2012 | 0.715 | 0.172 | 0.244 | 0.054 | 0.294 |
| 2013 | 0.705 | 0.193 | 0.238 | 0.040 | 0.286 |
| 2014 | 0.735 | 0.154 | 0.237 | 0.041 | 0.278 |
| 2015 | 0.752 | 0.163 | 0.221 | 0.039 | 0.288 |
| 2016 | 0.741 | 0.187 | 0.228 | 0.041 | 0.293 |
| 2017 | 0.735 | 0.175 | 0.190 | 0.046 | 0.252 |
| 2018 | 0.749 | 0.201 | 0.201 | 0.040 | 0.263 |
| 2019 | 0.731 | 0.176 | 0.200 | 0.042 | 0.268 |
| 2020 | 0.729 | 0.223 | 0.201 | 0.055 | 0.268 |
| mean of all years | 0.733 | 0.186 | 0.214 | 0.044 | 0.275 |

|  | FFS CARD | AMHSP CARD | FFS INMD | AMHSP INMD | Overall |
| --- | --- | --- | --- | --- | --- |
| 2012 | 0.383351 | 0.0110545 | 0.583101 | 0.0224935 | 0.294136 |
| 2013 | 0.375592 | 0.0120219 | 0.596084 | 0.0163024 | 0.285841 |
| 2014 | 0.394001 | 0.00950586 | 0.625256 | 0.0144227 | 0.278417 |
| 2015 | 0.410138 | 0.00812523 | 0.568766 | 0.0129706 | 0.287598 |
| 2016 | 0.409418 | 0.00821476 | 0.569459 | 0.0129089 | 0.292738 |
| 2017 | 0.393309 | 0.00817145 | 0.58449 | 0.0140302 | 0.252266 |
| 2018 | 0.374145 | 0.0114304 | 0.603558 | 0.0108669 | 0.262766 |
| 2019 | 0.375939 | 0.00668372 | 0.605496 | 0.0111395 | 0.267707 |
| 2020 | 0.389212 | 0.0078719 | 0.587977 | 0.0131497 | 0.267549 |

**2. We also examined whether the ORs change over time. We did this by rerunning the analysis in Figure 3 for the first two years of the cohort (2011 and 2012) and the last two years of the cohort (2018 and 2019). The ORs for physician payment model (our main exposure variable) remain very consistent over time when controlling for patient household income, patient age, patient sex, patient number of cardiovascular comorbidities, physician gender, physician age, and geographic region. This is reassuring. Given that we have 4 figures and 3 tables, we have decided not to include this information in the paper, but wanted to present this for your information.**

| **OR’s for Physician payment plan and specialty ***  Reference case: FFS cardiologist | | |
| --- | --- | --- |
|  | 2011-2012 | 2018-2019 |
| Salary-based cardiologist | 0.13  (0.07-0.25) | 0.13  (0.07-0.26) |
| Salary-based internal medicine | 0.03  (0.02-0.05) | 0.02  (0.01-0.04) |
| FFS internal medicine | 0.07  (0.05-0.11) | 0.07  (0.04-0.10) |

* **controlling for patient household income, patient age, patient sex, patient number of cardiovascular comorbidities, physician gender, physician age, and geographic region**

5.2 The interpretation of ORs should be supported with absolute risk or test rate examples to enhance real-world understanding.

**Thank you. This is a good suggestion, and does enhance real-world understanding. We considered various scenarios to estimate the expected likelihood of cardiac testing for the four different types of physicians, keeping patient characteristics the same, and using the regression coefficients from Figure 3. We consider four scenarios:**

**Scenario 1: Male; income quintile “3”; patient age 41-60; fewer than 3 patient non-cardiac comorbidities; physician gender=man; physician age 50; physician zone – urban 1**

**scenario 2: MALE; income quintile "3"; patient age 41-60; fewer than 3 patient non-cardiac comorbidities; physician gender=WOMAN; physician age 50; physician zone – urban 1**

**SCENARIO 3: Male; income quintile “3”; patient age 41-60; fewer than 3 patient non-cardiac comorbidities; physician gender=man; physician age 50; physician zone – rural 3**

**SCENARIO 4: FEMALE; income quintile "3"; patient age 41-60; fewer than 3 patient non-cardiac comorbidities; physician gender=MAN; physician age 50; physician zone – urban 1**

|  | Unadjusted likelihood of testing | scenario 1 – predicted risk of testing | scenario 2 – predicted risk of testing | scenario 3- predicted risk of testing | scenario 4 – predicted risk of testing |
| --- | --- | --- | --- | --- | --- |
| salary-based internal medicine specialists | 4.4% | 9.0% | 6.7% | 12.1% | 7.8% |
| FFS internal medicine specialists | 21.4% | 14.8% | 11.1% | 19.5% | 12.9% |
| salary-based cardiologists | 18.6% | 32.6% | 25.9% | 40.3% | 29.2% |
| FFS cardiologist | 73.3% | 64.1% | 56.3% | 71.3% | 60.4% |

**We have added this information to the paper.**

5.3 No subgroup results by sex, rurality, or age are discussed — such insights could enrich interpretation.

**Thank you. Although these are presented in Figure 2, the reviewer is correct that we inadvertently did not discuss this, and have done so now.**

5.4 Specialty comparisons could be disaggregated further for clarity (e.g., GP vs internist vs cardiologist).

**We apologize for any confusion. Our study focuses on visits by specialists (internists or cardiologists) – we have not included information on primary care visits. We have clarified this within the paper, adding specialists to clarify.**

5.5 A map or graph showing regional imaging variation would be informative. –

**We considered adding in GIS mapping, but again we feel this would dilute the message of the paper. We do show geographic variation in Table 2, and now highlight this in the text of the article.**

6. Discussion
6.1 The discussion is balanced but too narrowly scoped.

**Thank you. We have broadened the discussion.**

6.2 Future research directions are not addressed. Consider suggesting:

Multi-jurisdictional comparisons

Studies with clinical data to assess appropriate vs inappropriate imaging

Implications for payment reform or low-value care monitoring

**Thank you – we have added this.**

6.3 The conclusion line is overly technical: “Physician payment models and specialty are strongly associated with receipt of imaging…” Consider rephrasing for global readability, e.g.:
“How physicians are paid appears to influence imaging use patterns, even in low-risk patients. This has implications for health systems aiming to reduce low-value care.”

**Thank you. We have reframed this.**

7. Global Relevance and Framing
7.1 The framing leans heavily on Alberta/Canada context. Consider:

Generalizing findings into broader health economics or system design language -  Citing international frameworks (e.g., OECD health data, Choosing Wisely Global)
7.2 While local details are critical, striking a balance between specificity and transferability would increase the paper’s reach and relevance.

**Thank you. We have significantly reworked the discussion section.**

This manuscript is well-conceived and methodologically sound but would benefit from revisions that strengthen its clarity, expand its global framing, and deepen result interpretability. Addressing the issues outlined above will improve the paper . all the best

**Thank you. We believe that the extensive changes we have undertaken, based on your excellent comments, have significantly improved our paper.**
